# Prehospital Use of Lights and Sirens in Stroke Is Associated With Faster Door-to-CT Times but Not Door-to-Thrombolysis or Door-to-Endovascular Therapy Times

**DOI:** 10.1016/j.acepjo.2026.100415

**Published:** 2026-05-05

**Authors:** Courtney D. Wham, Daria Nicke, Ian S. Espinoza, Logan Murphy, Geoffrey Sowitz, Stefan Sillau, Andra M. Farcas, Michelle Leppert, Elizabeth Molina Kuna, Layne Dylla, IMPACT Investigators

**Affiliations:** 1 Department of Emergency Medicine, University of Colorado School of Medicine, Aurora, Colorado, USA; 2 Paramedic Division, Denver Health Medical Center, Denver, Colorado, USA; 3 Department of Neurology, University of Colorado School of Medicine, Aurora, Colorado, USA; 4 Department of Health Systems, Management, and Policy, Colorado School of Public Health, Aurora, Colorado, USA; 5 Department of Emergency Medicine, Yale School of Medicine, New Haven, Connecticut, USA

## Abstract

**Objectives:**

Lights and sirens (L&S) during Emergency Medical Services (EMS) transport may shorten transport times and improve outcomes in acute stroke but carry significant roadway risk. This study assesses L&S and in-hospital stroke care, including door-to-CT, door-to-intravenous thrombolysis (IVT), door-to-endovascular thrombectomy (EVT), and discharge disposition.

**Methods:**

This retrospective cohort study analyzed data from 2020 to 2022, including adult patients identified by EMS as having suspected stroke and who had a final diagnosis of stroke or transient ischemic attack. Data were collected from the IMPACT network (70 EMS agencies, 30 hospitals, 2 states). Multivariable regression modeled the association between L&S and door-to-CT, door-to-IVT, and door-to-EVT times, controlling for confounders. Logistic regression modeled the association between L&S and discharge disposition, adjusting for confounders and in-hospital intervention.

**Results:**

Of 3,161 patients, 75.4% (n = 2384) were transported with L&S. Transport time with L&S was shorter than without (geometric mean 8.9 minutes [95% CI 8.7-9.1] vs 11.6 minutes [95% CI 11.0-12.1], *p* < .001). L&S were associated with shorter door-to-CT (ratio estimate, 0.51; 95% CI, 0.46-0.56), but not with door-to-IVT (ratio estimate, 0.94; 95% CI, 0.82-1.08) nor door-to-EVT (ratio estimate, 1.03; 95% CI, 0.76-1.39). L&S were not associated with improved discharge outcomes.

**Conclusion:**

While L&S were associated with faster door-to-CT times, they did not shorten time to IVT or EVT or improve discharge outcomes. These findings suggest that perceived urgency of L&S may prompt quicker imaging, but transport time savings are minimal and may not improve outcomes. Alternative EMS prenotification and hospital activation strategies merit consideration.

**The Bottom Line**

Lights and sirens signal urgency and are assumed to expedite definitive care, but they nearly double the risk of a crash and offer only minimal time savings that may not improve outcomes. In time-sensitive conditions like stroke, it is unclear if even minor time differences are clinically relevant.This retrospective analysis of 3,161 patients with EMS-identified suspected stroke and confirmed hospital diagnoses across 70 EMS agencies and 30 hospitals evaluated the impact of lights and sirens on patient outcomes. Lights and sirens were associated with shorter door-to-CT times but not with improvements in time to thrombolysis, thrombectomy, or discharge outcomes.

## 1 Introduction

### 1.1 Background

Emergency warning signals have long been a required component of Emergency Medical Services (EMS) to help ambulances travel more quickly through traffic. It is often assumed that faster transport to definitive care improves patient outcomes and that the use of emergency signals will facilitate this. However, studies have shown only marginal time differences when EMS uses lights and sirens (L&S).^1^, ^2^, ^3^ This time difference has not consistently resulted in improved patient outcomes.

While rapid response to the scene may facilitate faster stabilization of the patient by prehospital clinicians, many life-saving interventions are only available in the hospital setting. In acute ischemic stroke, intravenous thrombolytics (IVT) and endovascular thrombectomy (EVT) are the only acute interventions available and can only be performed in the hospital or within a mobile stroke unit. These treatments can lead to meaningful patient recovery even when administered within 4.5 hours after stroke onset for IVT or 24 hours for EVT.^4^^,^^5^ In cases of thrombectomy, every minute faster to EVT means an additional week of functional independence.^6^ Thus, even small differences in time to treatment may improve outcomes when minutes count.

However, the use of L&S is not without risk. The rate of ambulance crashes more than doubles when L&S is used.^7^ There is also a “wake effect,” where crashes occur as traffic resumes after an ambulance passes. About 6500 ambulance-related crashes occur annually in the USA, with the wake effect causing roughly 5 times more incidents than those involving ambulances directly.^8^

### 1.2 Importance

Prior research suggests that prehospital interventions can influence in-hospital care, including door-to-computed tomography (CT) times and discharge disposition.^9^ It remains unknown if the use of L&S improves outcomes for patients with a stroke. With a transport mode that confers risk, research is needed to determine if the risk is worth the benefit to these patients.

### 1.3 Goal of this Investigation

The use of L&S within EMS may be necessary for patients with immediate life threats, despite increased risk.^7^ The objective of this study is to determine if L&S in acute stroke is associated with improved in-hospital stroke care and patient outcomes.

## 2 Methods

### 2.1 Study Design and Population

This retrospective cohort analysis included adults (aged 18 years and older) who were identified by EMS as having either a primary or secondary impression of stroke, cerebrovascular accident (CVA), or transient ischemic attack (TIA), or who were activated as a “stroke alert” from the prehospital setting between January 1, 2020, and December 31, 2022. Of these patients, only those with a final hospital diagnosis of CVA or TIA were included in the final population. The study included patients from a mix of 70 private and fire-based EMS agencies and 30 associated hospitals. Interfacility transfers were excluded. Participating hospitals included both academic and community hospitals with varying stroke center certifications, including some non–stroke-certified institutions. However, there were no thrombectomy-capable stroke centers.

### 2.2 Measurements

Data were collected using standardized case report forms created in Research Electronic Data Capture (REDCap), a HIPAA-compliant program. All EMS agency variables and data extracted directly from the electronic medical record for hospitals that were not recognized stroke centers were entered directly into REDCap, and data were validated using internal REDCap validation rules. Additional in-hospital data were extracted from local Get with the Guidelines-Stroke (GWTG-S) registries for any hospital that was a recognized stroke center. Data extracted from the local GWTG-S registry included age, sex, race, ethnicity, past medical history, time to CT, time to IVT, time to EVT, National Institutes of Health Stroke Scale (NIHSS) score, and discharge disposition. Prehospital data included age, sex, race, ethnicity, computer-aided dispatch times, primary and secondary impressions, vital signs, exam findings, interventions performed prior to hospital arrival, and compliance with American Heart Association (AHA) recommendations for prehospital care of patients with suspected stroke.^10^, ^11^, ^12^, ^13^ These AHA guidelines include (1) documentation of a stroke scale, (2) prenotification of receiving hospital when a stroke is suspected, (3) obtaining a blood glucose measurement, (4) performing a 12-lead electrocardiogram, (5) provision of supplemental oxygen for hypoxia (pulse oximetry less than 94%), (6) documentation of LKW time, (7) less than 2 minutes from dispatch to being en route, and (8) less than 15 minutes on-scene time. Data were extracted by abstractors who underwent standardized training with a test set of at least 30 charts. If the abstractor was not at least 90% accurate, they underwent additional training until a minimum 90% accuracy was achieved.

### 2.3 Data Analyses

Descriptive statistics characterized the cohort, reporting the number and proportion for categorical variables and the mean with standard deviation for continuous variables, or the geometric mean with 95% upper and lower confidence limits and/or median with interquartile range (IQR) for right-skewed continuous variables. Univariate analysis was performed using a chi-square for categorical variables and Wilcoxon/Kruskal-Wallis test for continuous variables on the logarithmic scale for right-skewed continuous variables. A multivariable, multinomial logistic regression model was used to test the association between transportation with L&S and patient discharge disposition, while controlling for predetermined factors that influence discharge disposition (sex, age, initial NIHSS, presence of any cardiovascular risk factor, receipt of IVT or EVT, receiving facility stroke certification level, type of EMS agency based on rural-urban commuting area score [rural, suburban or urban], time between LKW and ED arrival) and transport time.^14^ Multivariable linear regression models with a random effect for hospital tested the association between transport with L&S and door-to-CT time and door-to-IVT time, while controlling for predetermined factors that influence door-to times (sex, age, initial NIHSS, receiving facility capabilities, documentation of a stroke scale, documentation of a blood glucose level, documentation of LKW, completion of EMS prenotification of suspected stroke) and transport time. These components are known EMS care components associated with shortened door-to-CT times.^9^ For the multivariable regression model for door-to-EVT time, the receiving facility capability was omitted, as EVT could only be performed at a comprehensive stroke center. In regression models including transport time as a covariate, observations with values in the greater than 99th percentile (more than 37 minutes, n = 37) were omitted, as these outliers could distort the model fit. Time variables were right-skewed, so medians were used for descriptive analyses and log transformation was applied for modeling to enable conventional regression, with results reported as geometric means after back-transformation. Analyses were performed on an available case basis. All analyses were performed using SAS 9.4 software (SAS Institute Inc, Cary, NC).

### 2.4 Ethics Approval

This study was approved by the Colorado Multi-Institutional Review Board (COMIRB#23-1035). It adhered to the Code of Ethics of the World Medical Association, Declaration of Helsinki.

## 3 Results

### 3.1 Subject Demographics and EMS Care

Within the study period, 3,161 patient encounters were identified as meeting the inclusion and exclusion criteria. Of these encounters, 75.4% (n = 2,384) were transported with L&S, and 24.6% (n = 776) were transported without L&S. The overall study group was 50.3% (n = 1588) female, 87.3% (n = 2,072) White, 5.9% (n = 141) Black, 1.9% (n = 46) Asian, and 4.8% (n = 115) other (Table 1). There were 5.7% (n = 180) of subjects identified as Hispanic or Latino. Those transported with and without L&S did not differ by sex, race, or ethnicity. Those transported with L&S were younger (median age 75.0 years, IQR 64.0-84.0 years) than those transported without L&S (median age 76.0 years, IQR 65.0-85.0 years, *p* = .02). The median NIHSS score for patients transported with L&S was higher (8, IQR 3-16) than those for patients transported without L&S (3, IQR 1-8, *p* < .001). The total transport time for patients transported without L&S was 2.7 minutes longer than for patients transported with L&S (geometric mean 11.6 minutes [95% CI 11.0-12.1] vs 8.9 minutes [95% CI 8.7-9.1], *p* < .001).

**Table 1:** Patient characteristics.

Characteristic	Lights and sirens (n = 2384)	No lights and sirens (n = 776)
**Age (years)**,∗ median (IQR); geometric mean (95% upper and lower limits)	75.0 (64.0-84.0); 73.0 (72.4, 73.6)	76.0 (65.0-85.0); 74.3 (73.3, 75.3)
**Sex, n (%)**		
Female	1186 (49.8%)	402 (51.8%)
Male	1198 (50.3%)	374 (48.2%)
**Race**^a^**, n (%)**		
White	1487 (86.7%)	585 (88.8%)
Black	109 (6.4%)	32 (4.9%)
Asian	32 (1.9 %)	14 (2.1%)
Other	87 (5.1%)	28 (4.2%)
**Ethnicity, n (%)**		
Hispanic/Latino	135 (5.7%)	45 (5.8%)
Not Hispanic/Latino	2249 (94.3%)	731 (94.2%)
**Any cardiovascular comorbidity, n (%)**	1994 (83.6%)	611 (78.7%)
**Initial NIHSS,**∗ median (IQR)	8 (IQR 3-16)	3 (IQR 1-8)
**Transport time (minutes)**,∗ median (IQR); geometric mean (95% upper and lower limits)	9.0 (6.0-13.0); 8.9 (8.7, 9.1)	12.5 (8.0-18.0); 11.6 (11.0, 12.1)
**Receiving hospital certification level, n (%)**		
Not certified	138 (5.8%)	127 (16.4%)
Acute stroke ready or primary stroke center	915 (38.4%)	294 (37.9%)
Comprehensive stroke center	1332 (55.9%)	355 (45.8%)
**Time from LKW to door (minutes)**∗^,^^b^—median (IQR); geometric mean (95% upper and lower limits)	87 (45.0-301.0); 117.1 (111.1, 123.5)	166.0 (67.0-607.0);189.5 (170.5, 210.6)
**Any acute intervention, n (%)**	736 (30.9%)	92 (11.9%)
**Discharge Disposition,∗^,^**^c^**n (%)**		
Home	882 (40.1%)	335 (46.7%)
Need of care	938 (42.6%)	266 (37.1%)
Hospice or expired	382 (17.4%)	117 (16.3%)
**Door-to-CT time (minutes)**,∗^,^^d^ median (IQR); geometric mean (95% upper and lower limits)	10.0 (7.0-13.0); 9.4 (9.1, 9.8)	20.0 (10.0-58.0); 22.8 (21.0, 24.7)
**Door-to-IVT time (minutes)**,∗ median (IQR); geometric mean (95% upper and lower limits)	36.0 (27.0-50.0); 37.4 (35.8, 39.0)	45.0 (33.0-65.0); 46.3 (41.0, 52.1)
**Door-to-EVT time (minutes)**, median (IQR); geometric mean (95% upper and lower limits)	92.0 (81.0-118.0); 97.0 (91.5, 102.8)	84.5 (63.0-107.0); 89.6 (68.7, 117.0)

∗*p* < .05 by chi-square or Wilcoxon rank-sum test of those with vs without lights and sirens.

a Missingness: ^a^race =786 (24.9%) missing encounters, ^b^time between LKW and arrival - 825 (26.1%) missing encounters, ^c^discharge disposition - 240 (7.6%) missing encounters, ^d^door-to-CT time - 100 (3.2%) encounters, missing values were excluded from analysis and modeling.

In terms of compliance with AHA recommendations for prehospital care of patients with suspected stroke (Table 2), a higher proportion of encounters transported with L&S were fully compliant with AHA guidelines (12.8%, n = 306) compared to those transported without L&S (5.3%, n = 41, p < .001). This included more frequent EMS to hospital prenotification (73.3%, n = 1,747 vs 32.1%, n = 249; *p* < .001), more frequent documented LKW time (81%, n = 1,930 vs 67.9%, n = 527, *p* < .001), and more frequent compliance with a goal of less than 15 minutes on-scene time (61.1%, n = 1,456 vs 38.7%, n = 300; *p* < .001) among those transported with L&S compared to those transported without.

**Table 2:** Proportion of EMS encounters compliant with AHA recommendations.

Recommendation^a^	Lights and Sirens N (%)	No Lights and Sirens N (%)
EMS on scene time ≤ 15 minutes∗	1456 (61.1%)	300 (38.7%)
Dispatch to EMS en route ≤ 90 seconds	1907 (80.0%)	617 (79.5%)
Documentation of a LKW time∗	1930 (81.0%)	527 (67.9%)
Documented BGL∗	2050 (86.0%)	637 (82.1%)
Documented 12-lead ECG∗	1866 (78.3%)	534 (68.8%)
Documentation of a validated stroke scale∗	1815 (76.1%)	524 (67.5%)
EMS prenotification to receiving facility∗	1747 (73.3%)	249 (32.1%)
Provision of supplemental oxygen only for SpO2 <94%	1284 (53.9%)	428 (55.2%)
Complete AHA recommendation compliance∗	306 (12.8%)	41 (5.3%)

BGL, blood glucose level; ECG, electrocardiogram; LKW, last known well time; SpO2, pulse oximetry; EMS, emergency medical services; AHA, American Heart Association.

∗Chi-square *p* value <.05.

a Any item that was not documented was considered not performed.

### 3.2 Use of L&S is Associated With Reduced Door-to-CT Time

After controlling for known confounders, transportation with L&S was associated with reduced door-to-CT time (ratio estimate 0.51 [0.46-0.56]) (Supplemental Table 1a). The resulting adjusted geometric mean door-to-CT time for those transported with L&S was 10.3 minutes (95% CI 8.1-13.0 minutes) and was 20.3 minutes (95% CI 16.0-25.9 minutes) for those transported without L&S.

Furthermore, increased NIHSS, EMS documented LKW time, hospital prenotification of suspected stroke, and shorter transport times were also associated with reduced door-to-CT times (Supplemental Table 1a). Prenotification of a suspected stroke patient resulted in a 31% reduction in door-to-CT time amongst those with prenotification compared to those without prenotification (Supplemental Table 1a). EMS documentation of an LKW time was associated with a 9% reduction in door-to-CT time compared to those encounters in which the LKW was not documented. For every unit increase in NIHSS score, there was a less than 1% decrease in door-to-CT time. For every additional minute of EMS transport time (the time from scene departure to arrival at the hospital), there was a 1% increase in door-to-CT time. These small differences in door-to-CT times should be interpreted with caution, given they represent only seconds of time saved.

### 3.3 Use of L&S Is Not Associated With Reduced Door-to-IVT Time

Within the overall cohort, 18.3% (n = 579) of patients received IVT. Of these, 88.6% (n = 513) were transported with L&S, and 11.4% (n = 66) were transported without L&S. When controlling for confounders, transport with L&S compared to transport without L&S was not associated with reduced door-to-IVT time (ratio estimate 0.94 [95% CI 0.82-1.08]) (Supplemental Table 1b). Transport time was also not associated with door-to-IVT time (ratio estimate 1.00 [95% CI 0.99-1.01]). The adjusted geometric mean door-to-IVT time for those transported with L&S was 45.1 minutes (95% CI 40.0-50.8 minutes) compared to 48.0 minutes (95% CI 41.2-55.8 minutes) for those transported without L&S.

### 3.4 Use of L&S is Not Associated With Reduced Door-to-EVT Time

Within the overall cohort, 5.58% (n = 182) patients received endovascular therapy with 77.47% (n = 141) of those patient encounters transported using L&S and 7.79% (n = 14) without using L&S. When controlling for confounders, neither transport with L&S nor transport time were associated with a reduced door-to-EVT time (L&S - ratio estimate 0.94 [95% CI 0.82-1.08]; transport time - ratio estimate 1.00 [95% CI 0.99-1.01]) (Supplemental Table 1c). The adjusted geometric mean door-to-EVT time for those transported with L&S was 104.2 minutes (95% CI 76.8-141.5 minutes) compared to 101.3 minutes (95% CI 69.7-147.3 minutes) for those transported without L&S.

### 3.5 Use of L&S is Not Associated With Differences in Patient Outcomes

Those transported with L&S differed in their ultimate discharge disposition compared to those transported without L&S (Table 1). The adjusted odds of discharge home or in need of care compared to death or hospice were similar among patients transported with and without L&S (ratio estimate 1.27 [95% CI 0.83-1.93] and 1.40 [(95% CI 0.94-2.10], respectively) (Supplemental Table 2a). Additionally, those patients transported with and without L&S had similar odds of being discharged in need of care as opposed to home (ratio estimate 1.11 [95% CI 0.84-1.46]) (Supplemental Table 2b). Transport time was also not associated with a difference in odds of being discharged home or in need of care compared to death or hospice or being discharged in need of care compared to home. However, transport to a primary stroke center instead of an unrecognized hospital was associated with 19.43 (95% CI 8.31-45.46) times the odds of discharge to home instead of death or hospice.

## 4 Limitations

There were several limitations in this study. Inclusion of data from many different EMS agencies and hospitals results in system-based variability that could influence the effects of L&S on in-hospital care. It is possible that the heterogeneity of the cohort may confer limited generalizability to a specific system. However, it may also be a strength, lending credence to the study’s generalizability, as it reflects multiple systems and the practice variation that exists between them. Furthermore, this study relied on data obtained and recorded by EMS agencies and hospitals, which resulted in missing data, especially with regard to discharge disposition (∼7.6% missing). However, the large sample size helps to overcome this issue. Additionally, there were also low outcomes of interest in the cohorts receiving IVT or EVT, as compared to the overall cohort, thereby limiting the ability to detect a small but significant association between transport with L&S and time to treatment. The findings should be interpreted with caution, given the small sample size within these cohorts. Additionally, while NIHSS was used to control for the severity of the stroke, there may be residual confounding related to clinical judgment of stroke severity not reflected by the NIHSS which could affect transport decision and hospital workflow activation. Transport times greater than the 99th percentile (>37 minutes) were also excluded to improve model stability, which may have removed clinically meaningful observations, especially in rural settings. The removal of these 37 cases of the 3732 total and the absence of a sensitivity analysis may limit the interpretation of these findings across a full spectrum of prehospital systems. Despite these limitations, this study provides insight into the association between L&S and patient care and outcomes in acute stroke. However, implications and recommendations regarding the use of L&S in the prehospital setting are beyond the scope of this study.

## 5 Discussion

This retrospective analysis of patients with prehospital provider impression of suspected stroke and a final diagnosis of stroke or TIA found that the use of L&S by EMS was associated with shorter door-to-CT times. However, there was no significant difference in time to IVT, time to EVT, or discharge disposition after controlling for other known factors affecting these outcomes. The small differences in door-to-CT times associated with the use of L&S or other covariates may not be clinically significant.

This study found that transport with L&S, EMS prenotification, EMS documentation of LKW time, and EMS transport time were associated with shorter door-to-CT times, but clinical impact varied. EMS prenotification to the receiving hospital of concerns for a suspected stroke was associated with the largest reduction in door-to-CT time (31%). Of note, all agencies in this study provided some form of hospital prenotification for every patient, regardless of whether or not it was a formal “stroke alert” or the use of L&S. Use of L&S was left to provider discretion rather than protocol. Documentation of LKW time was the next strongest factor, associated with a 9% reduction in door-to-CT time. Stroke severity as indicated by NIHSS score had an even more limited impact, with each point increase having a 1% in decrease in door-to-CT time. Transport time also had minimal impact, with each minute saved regardless of L&S use translating to only about a 2-second reduction in door-to-CT time, which is unlikely to be clinically significant.

While prior research shows that prehospital care can influence outcomes, studies have found that L&S save only 1 to 3 minutes in transport times, which has not been associated with meaningful differences in clinical outcomes.^3^^,^^15^ The results presented here are also consistent with prior studies. Though minor time savings of L&S may not affect outcomes, the signal of urgency from L&S use may still play an important role in speeding initial hospital evaluation. EMS prenotification of suspected strokes has previously been associated with improved in-hospital care, including shorter time to CT and improved functional outcomes after hospitalization.^9^ During transport, prehospital clinicians often communicate directly with hospital staff, sharing key patient information before arrival, especially during transport with L&S, even without a formal stroke alert. This catalyzes a series of events within the hospital before the ambulance arrives and may facilitate streamlined care directly related to the urgency of the EMS call.

However, the primary concern with the use of L&S remains its impact on safety.^7^^,^^8^ Studies have shown an increase in ambulance collisions, more than doubling of transport-phase crashes with the use of L&S, which cause $500 million in damages annually.^7^^,^^16^ Approximately 60% of fatal ambulance collisions involve L&S, with most fatalities affecting the public, not EMS personnel.^17^ Despite known safety risks and minimal time savings, L&S remain widely used as emergency systems try to balance risk with potential benefit.^2^ Future approaches may focus on alternative communication strategies that preserve urgency without requiring L&S use, such as a more formalized universal prehospital stroke alert protocol. Significant variability between agencies exists regarding prehospital stroke protocols, and standardization of this process based on objective information from EMS crews could serve as a uniform solution to this issue without the need to invoke the preconceived biases associated with the use of L&S.

The results of this study warrant further investigation into the best situations for the use of L&S. When EMS providers transport using L&S, only a few minutes in transport time are saved, associated with only shortened door-to-CT times. Given the lack of association between use of L&S and door-to-treatment times, other key factors influencing in-hospital care and patient outcomes also deserve more focus. Both EMS prenotification and documentation of a LKW time were much stronger contributors to both door-to-CT and door-to-IVT times. EMS education could emphasize standardized prenotification, stroke screening and neurological assessment, and obtaining a LKW time to streamline in-hospital care. These approaches may help facilitate expeditious evaluation and treatment to improve patient outcomes while limiting the risks of L&S during prehospital transport.

## Author Contributions

Conceptualization: CW, LD, DN; Methodology and Data Collection: CW, LD, DN, IE, GS, LM; Formal Analysis and Interpretation: SS, LD, CW; Writing - Original Draft Preparation: CW, LM; Writing Review and Editing: CW, LD, CN, IE, SS, AD, ML, EMK, LM; Funding Aquisition: CW, LD

## Funding and Support

University of Colorado IMPACT Research Fund (anonymous donor); REDCap through NIH/NCATS Colorado CTSA Grant Number UM1 TR004399.

## Data Sharing Statement

The fully de-identified data that support the findings of this study are available from the corresponding author, [L.D.], upon reasonable request.

## Conflict of Interest

C.W. reports serving as a consultant for Analog Devices, Inc. in 2025, a company involved in the development of healthcare technology. This relationship is outside of the submitted work and is not ongoing. L.D. reports grant money to Yale University School of Medicine to conduct research conceived and sponsored by TETMedical, Inc. This relationship is outside of the submitted work. All other authors declare no conflicts of interest.

## Supplementary Materials

Supplementary Tables 1and 2
