# Supplementary material for: Prehospital Use of Lights and Sirens in Stroke Is Associated With Faster Door-to-CT Times but Not Door-to-Thrombolysis or Door-to-Endovascular Therapy Times
Source: J Am Coll Emerg Physicians Open. 2026 May 5;7(3):100415. doi: 10.1016/j.acepjo.2026.100415 (PMC13158396; doi:10.1016/j.acepjo.2026.100415)
Supplement: Supplementary Tables 1and 2 [file mmc1.docx]

| Supplemental Table 1a: Full GLMMIX Model for Door-to-CT | |
| --- | --- |
| Comparison | **Ratio Estimate (Lower - Upper CI)** |
| With vs Without Lights and Sirens | 0.51 (0.46 – 0.56) |
| PSC/ASRH vs Not Certified | 0.69 (0.41 – 1.17) |
| CSC vs Not Certified | 0.96 (0.52 – 1.79) |
| CSC vs PSC/ASRH | 1.39 (0.81 – 2.38) |
| Male vs Female | 0.97 (0.92 – 1.03) |
| Age (per year) | 1.00 (1.00 – 1.00) |
| NIHSS (per unit) | 1.00 (0.99 – 1.00) |
| Documented Stroke Scale | 0.94 (0.87 – 1.01) |
| BGL obtained | 1.03 (0.94 – 1.12) |
| Documented LKW | 0.91 (0.85 – 0.98) |
| Prenotification to Receiving Hospital of Suspected Stroke | 0.69 (0.64 – 0.74) |
| Transport Time (per minute) | 1.01 (1.00 – 1.01) |

| Supplemental Table 1b: Full GLMMIX Model for Door-to-IVT | |
| --- | --- |
| Comparison | **Ratio Estimate (Lower - Upper CI)** |
| With vs Without Lights and Sirens | 0.94 (0.82 – 1.08) |
| PSC/ASRH vs Not Certified | 0.76 (0.59 – 0.94) |
| CSC vs Not Certified | 0.71 (0.54 – 0.94) |
| CSC vs PSC/ASRH | 0.94 (0.76 – 1.16) |
| Male vs Female | 0.94 (0.87 – 1.02) |
| Age (per year) | 1.00 (1.00 – 1.00) |
| NIHSS (per unit) | 1.00 (0.99 -1.00) |
| Documented Stroke Scale | 0.93 (0.84 – 1.03) |
| BGL obtained | 0.93 (0.82 – 1.06) |
| Documented LKW | 0.99 (0.78 – 0.95) |
| Prenotification to Receiving Hospital of Suspected Stroke | 0.86 (0.78 – 0.95) |
| Transport Time (per minute) | 1.00 (0.99 – 1.01) |

| Supplemental Table 1c: Full GLMMIX Model for Door-to-EVT | |
| --- | --- |
| Comparison | **Ratio Estimate (Lower - Upper CI)** |
| With vs Without Lights and Sirens | 1.03 (0.76 – 1.39) |
| Male vs Female | 1.02 (0.91 – 1.13) |
| Age (per year) | 1.00 (1.00 – 1.00) |
| NIHSS (per unit) | 1.00 (0.99 – 1.01) |
| Documented Stroke Scale | 0.97 (0.85 – 1.12) |
| BGL obtained | 1.10 (0.94 – 1.28) |
| Documented LKW | 0.92 (0.79 – 1.06) |
| Prenotification to Receiving Hospital of Suspected Stroke | 0.97 (0.86 – 1.09) |
| Transport Time (per minute) | 1.01 (1.00 – 1.02) |

Abbreviations: ASRH – acute stroke ready hospital, BGL – blood glucose level, CI – confidence interval, CSC – comprehensive stroke center, NIHSS – National Institutes of Health Stroke Scale, PSC – primary stroke center.

| Supplemental Table 2a: GLIMMIX Procedure – Discharge Disposition Compared to Expired or Hospice | | |
| --- | --- | --- |
| Label | **Discharge Disposition vs Expired** | **Odds Ratio (95% Cl)** |
| With vs Without Lights and Sirens | Home | 1.27 (0.83 -1.93) |
|  | Need of Care | 1.40 (0.94 – 2.10) |
| PSC vs None | Home | 19.43 (8.31 – 45.46) |
|  | Need of Care | 5.14 (2.51 – 10.53) |
| CSC vs None | Home | 15.25 (6.48 – 35.92) |
|  | Need of Care | 3.72 (1.80 – 7.69) |
| CSC vs ASRH/PSC | Home | 0.78 (0.54 – 1.13) |
|  | Need of Care | 0.72 (0.51 – 1.03) |
| Suburban vs Urban | Home | 0.67 (0.39 – 1.17) |
|  | Need of Care | 0.64 (0.38 – 1.08) |
| Rural vs Urban | Home | 0.59 (0.29 – 1.22) |
|  | Need of Care | 0.59 (0.30 – 1.15) |
| Rural vs Suburban | Home | 0.88 (0.38 – 2.01) |
|  | Need of Care | 0.92 (0.43 – 1.98) |
| Male vs Female | Home | 0.80 (0.59 – 1.09) |
|  | Need of Care | 0.69 (0.51 – 0.92) |
| Age per year | Home | 0.95 (0.93 – 0.96) |
|  | Need of Care | 0.95 (0.94 – 0.96) |
| Any CV Risk Factor | Home | 0.94 (0.58 – 1.53) |
|  | Need of Care | 1.09 (0.69 – 1.72) |
| NIHSS per Unit | Home | 0.81 (0.79 – 0.83) |
|  | Need of Care | 0.90 (0.89 – 0.92) |
| Time from LKW to Arrival (per minute) | Home | 1.00 (1.00 – 1.00) |
|  | Need of Care | 1.00 (1.00 – 1.00) |
| Any Intervention | Home | 1.77 (1.22 – 2.57) |
|  | Need of Care | 1.54 (1.10 – 2.18) |
| Total Transport Time (per minute) | Home | 1.00 (0.98 – 1.03) |
|  | Need of Care | 1.01 (0.98 – 1.04) |

| Supplemental Table 2b: GLIMMIX Procedure – Discharge Disposition Compared to Home | | |
| --- | --- | --- |
| Label | **Discharge Disposition vs Home** | **Odds Ratio (95% Cl)** |
| With vs Without Lights and Sirens | Hospice/Expired | 0.79 (0.52 – 1.21) |
|  | Need of Care | 1.11 (0.84 – 1.46) |
| PSC vs None | Hospice/Expired | 0.05 (0.02 – 0.12) |
|  | Need of Care | 0.26 (0.13 – 0.54) |
| CSC vs None | Hospice/Expired | 0.07 (0.03 – 0.15) |
|  | Need of Care | 0.24 (0.12 – 0.50) |
| CSC vs ASRH/PSC | Hospice/Expired | 1.27 (0.88 – 1.84) |
|  | Need of Care | 0.92 (0.74 – 1.16) |
| Suburban vs Urban | Hospice/Expired | 1.49 (0.85 – 2.59) |
|  | Need of Care | 0.95 (0.66 – 1.38) |
| Rural vs Urban | Hospice/Expired | 1.69 (0.82 – 3.51) |
|  | Need of Care | 0.99 (0.57 – 1.74) |
| Rural vs Suburban | Hospice/Expired | 1.14 (0.50 – 2.61) |
|  | Need of Care | 1.05 (0.56 – 1.95) |
| Male vs Female | Hospice/Expired | 1.25 (0.91 – 1.71) |
|  | Need of Care | 0.86 (0.70 – 1.05) |
| Age per year | Hospice/Expired | 1.06 (1.04 – 1.07) |
|  | Need of Care | 1.01 (1.00 – 1.01) |
| Any CV Risk Factor | Hospice/Expired | 1.07 (0.65 – 1.73) |
|  | Need of Care | 1.16 (0.85 – 1.58) |
| NIHSS per Unit | Hospice/Expired | 1.24 (1.21 – 1.27) |
|  | Need of Care | 1.12 (1.10 – 1.14) |
| Time from LKW to Arrival (per minute) | Hospice/Expired | 1.00 (1.00 – 1.00) |
|  | Need of Care | 1.00 (1.00 – 1.00) |
| Any Intervention | Hospice/Expired | 0.56 (0.39 – 0.82) |
|  | Need of Care | 0.87 (0.69 – 1.11) |
| Total Transport Time (per minute) | Hospice/Expired | 1.00 (0.97 – 1.02) |
|  | Need of Care | 1.00 (0.98 – 1.02) |

Abbreviations: ASRH – acute stroke ready hospital, BGL – blood glucose level, CI – confidence interval, CSC – comprehensive stroke center, NIHSS – National Institutes of Health Stroke Scale, PSC – primary stroke center.
