# Supplementary material for: Prehospital Use of Lights and Sirens in Stroke Is Associated With Faster Door-to-CT Times but Not Door-to-Thrombolysis or Door-to-Endovascular Therapy Times
Source: J Am Coll Emerg Physicians Open. 2026 May 5;7(3):100415. doi: 10.1016/j.acepjo.2026.100415 (PMC13158396; doi:10.1016/j.acepjo.2026.100415)
Supplement: Supplementary File [file mmc2.docx]

**Supplement 3a: The Impact Investigators**

**AdventHealth**

*Denver/Boulder Area, Colorado*

Danielle Hagedorn, MBA, BSN, RN, CPHQ

**AMR El Paso County**

Colorado Springs, Colorado

**Arvada Fire Protection District**

*Arvada, Colorado*

Dave Mitchell, BA, NRP

Robert Putfark, NRP

**Aurora Fire Rescue**

*Aurora, Colorado*

Brandon Sauer, NRP, CFO

Eric Hill, MD, FACEP, FAEMS

**Bennett-Watkins Fire Rescue**

*Bennett, Colorado*

Josie Fischer, NRP

**Boulder Community Health**

*Boulder, Colorado*

Bob Deering, RN

Rachel Glantz NRP, MEd

**Boulder Rural Fire Rescue**

*Boulder, Colorado*

Stephanie Sovndal, NRP

**Brighton Fire Rescue District**

*Brighton, Colorado*

**Byers Fire Protection District**

*Byers, Colorado*

MacKenzie Schledorn-Rudden, NRP

**Chaffee County EMS**

*Salida, Colorado*

**City of Wray Ambulance**

*Wray, Colorado*

Steve Rydquist CC-P, FP-C

Rebecca Mayer CAC, EMT-I, RN

**Clear Creek EMS**

*Idaho Springs, Colorado*

Clark Church, BSc, NRP, FP-C

Aaron Crawley, AAB, NRP, FP-C

**Colorado Springs Fire Department**

*Colorado Springs, Colorado*

Mark Warth, BHS, NRP

Eric Wu, MD, FAEMS

**CommonSpirit Health - St. Anthony’s Hospital**

*Wheat Ridge, Colorado*

Matthew C. Loftspring, MD, PhD

Jennifer McMahon MS, BSN, RN-BC, SCRN

**CommonSpirit Health - St. Francis Hospital**

*Colorado Springs, Colorado*

Christina Williamson BSN, RN, CCRN

**Crested Butte Fire Protection District**

*Crested Butte, Colorado*

Robert Weisbaum BS, FP-C, MPO

**Custer County EMS**

*Silvercliff, Colorado*

**Delta County Hospital**

*Delta, Colorado*

Ramona L. Frazier, RN

Kayleigh Wright, RN, BSN, TCRN, PECC

**Denver Health Medical Center - Department of Neurology**

*Denver, Colorado*

Robert Pratt, MD

**Elk Creek Fire Protection District**

*Conifer, Colorado*

Lorie Hartley, NRP

**Federal Heights Fire Department**

*Federal Heights, Colorado*

**Fountain Fire Department**

*Fountain, Colorado*

Jared Cass, CFO, CEMSO

**Frederick-Firestone Fire District**

*Frederick, Colorado*

Jeremy Young, MS, EFO, CFO

**Gilpin County EMS**

*Blackhawk, Colorado*

**Grand County EMS**

*Granby, Colorado*

Erich Barber, BS, NRP

Austin Wingate, BS, CCEMT-P

**Grand Junction Fire Department**

*Grand Junction, Colorado*

Mark McIntire, NRP, FP-C

**Gunnison Valley Health**

*Gunnison, Colorado*

Christiana Evans, BSN, RN, CEN

**Gunnison Valley Health Paramedics**

*Gunnison, Colorado*

Nicholas Zuber, FP-C, DEM

**InterCanyon Fire Protection District**

*Morrison, Colorado*

Suzannah Epperson, RN, NRP, CP-C

**Intermountain Health - Lutheran Hospital**

*Wheat Ridge, Colorado*

Jessica Telesco, MS, AGACNP-BC, CNRN

**Intermountain Health - Platte Valley Hospital**

*Brighton, Colorado*

Jessica Mahoney MSN, RN, CCRP

**Intermountain Health - St Mary's Regional Hospital**

*Grand Junction, Colorado*

Meredith Jones, BSN, SCRN

**Longmont Fire Department**

*Longmont, Colorado*

**Monument Fire Department**

*Monument, CO*

Stephanie Botkin, NRP

**Montrose Fire Protection District**

*Montrose, Colorado*

Avery MacKenzie, MD

**Montrose Regional Health**

*Montrose, Colorado*

Avery MacKenzie, MD

Ross Orpet, MD

**Morgan County Ambulance Service**

*Fort Morgan, Colorado*

Travis W. Freeman, AAS, FP-C, NRP

**Mountain View Fire Rescue**

*Longmont, Colorado*

Paul Johnson, NRP

**Nederland Fire Protection District**

*Nederland, Colorado*

Conor Moran, NRP

**North Metro Fire Rescue District**

*Broomfield, Colorado*

Mark Daugherty, NRP

**Olathe Fire Protection District**

*Olathe, Colorado*

Ross Orpet, MD

**Ouray County EMS**

*Ouray, Colorado*

**Platteville-Gilcrest Fire Protection District**

*Platteville, Colorado*

Matt Concialdi, MS, CEMSO, NRP

**Platte Valley Ambulance Service**

*Brighton, Colorado*

William C. McNitt MD, FACEP

Michael Webster, NRP

**Red, White & Blue Fire Protection District**

*Breckenridge, Colorado*

C. Sam Smith MD, Maj, USAFR

**Security Fire Protection District**

*Colorado Springs, Colorado*

Aaron R. Williams, BAS, NRP, P-CC

**South Metro Fire Rescue**

*Centennial, Colorado*

Jonathan D. Apfelbaum, MD, FACEP, FAAEM

Jens Pietrzyk, MS, CFO, CEMSO, FP-C

**South Park Ambulance District**

*Fairplay, Colorado*

C. Sam Smith MD, Maj, USAFR

**Southwest Teller County EMS**

*Cripple Creek, Colorado*

**Stadium Medical**

*Denver, Colorado*

Meghan Adas, EMT-B

**Steamboat Springs Fire Rescue**

*Steamboat, Colorado*

Joe Oakland, NRP

**Strasburg Fire Protection District**

*Strasburg, Colorado*

MacKenzie Schledorn-Rudden, NRP

**Summit Fire & EMS**

*Frisco, Colorado*

Rick Ihnken, NRP

C. Sam Smith MD, Maj, USAFR

**Thompson Valley EMS**

*Loveland, Colorado*

Colt Sheek, NRP

**UCHealth Highlands Ranch Hospital**

*Highlands Ranch, CO*

Rachel Williams, BSN, RN, CEN

**UCHealth Memorial Hospital**

*Colorado Springs, CO*

Donna Stringer, BSN, RN, SCRN

**UCHealth North Region**

*Fort Collins/Loveland, Colorado*

Amanda Werner, BSN, RN, MEDSURG-BC, SCRN, ASC-BC

Melinda Tafoya BSN, RN, SCRN

**University of Colorado School of Medicine - Department of Neurology**

*Aurora, Colorado*

Kerri A. Jeppson, RN, BSN, SCRN

Brandy C. Ravare, RN, BSN

**Ute Pass Regional Health District**

*Woodland Park, Colorado*

Arren Gavin, EMT-B

**West Metro Fire Rescue**

*Lakewood, Colorado*

Todd Heinl, NRP, MPA, CFO, CEMSO

Reed Zachary Louderback, MD

Jacob Dueñas, BS

*University of Colorado School of Medicine*

*Aurora, Colorado*

Robert Frakes, NRP

*Denver, Colorado*

Rachel Konopen, MD

*Denver Health Medical Center, Emergency Medicine Residency*

*Denver, Colorado*

Monisha Lensink-Vasan, BA

University of Colorado School of Medicine

*Aurora, Colorado*

Madison Martz, MD

*University of Colorado School of Medicine*

*Aurora, Colorado*

James O’Connor, BS, FP-C

*University of Colorado School of Medicine*

*Aurora, Colorado*

Harshini Ranjit

*University of Colorado at Denver*

*Denver, Colorado*

Melissa Smith, MD, RN

*Northwestern Memorial Hospital, Department of Emergency Medicine*

*Chicago, Illinois*

Breanna Thrower, BA, EMT-B

*University of Colorado at Boulder, Department of Integrative Physiology*

*Boulder, Colorado*

Zachary Trottier, MD

*Mayo Clinic, Department of Neurology*

*Rochester, Minnesota*

Lisa Viltz, MD, MS

*Mayo Clinic, Department of Emergency Medicine*

*Rochester, Minnesota*

Angela Wright, MD, FAEMS

*University of Colorado School of Medicine, Department of Emergency Medicine*

*Aurora, Colorado*
